# Catalytic Antibodies in Bipolar Disorder: Serum IgGs Hydrolyze Myelin Basic Protein

**DOI:** 10.3390/ijms23137397

**Published:** 2022-07-02

**Authors:** Daria A. Kamaeva, Liudmila P. Smirnova, Svetlana N. Vasilieva, Daria V. Kazantseva, Alisa R. Vasilieva, Svetlana A. Ivanova

**Affiliations:** 1 Mental Health Research Institute, Tomsk National Research Medical Center of the Russian Academy of Sciences, 634014 Tomsk, Russia; lpsmirnova@yandex.ru (L.P.S.); vasilievasn@yandex.ru (S.N.V.); ivanovaniipz@gmail.com (S.A.I.); 2 Department of Biomedicine, Siberian State Medical University, 634055 Tomsk, Russia; dashka1745@mail.ru (D.V.K.); likenikitina2015@gmail.com (A.R.V.)

**Keywords:** bipolar disorder, catalytic IgG, abzymes, myelin, myelin basic protein, antibodies, humoral immunity

## Abstract

The pathogenesis of bipolar affective disorder is associated with immunological imbalances, a general pro-inflammatory status, neuroinflammation, and impaired white matter integrity. Myelin basic protein (MBP) is one of the major proteins in the myelin sheath of brain oligodendrocytes. For the first time, we have shown that IgGs isolated from sera of bipolar patients can effectively hydrolyze human myelin basic protein (MBP), unlike other test proteins. Several stringent criteria were applied to assign the studied activity to serum IgG. The level of MBP-hydrolyzing activity of IgG from patients with bipolar disorder was statistically significantly 1.6-folds higher than that of healthy individuals. This article presents a detailed characterization of the catalytic properties of MBP-hydrolyzing antibodies in bipolar disorder, including the substrate specificity, inhibitory analysis, pH dependence of hydrolysis, and kinetic parameters of IgG-dependent MBP hydrolysis, providing the heterogeneity of polyclonal MBP-hydrolyzing IgGs and their difference from canonical proteases. The ability of serum IgG to hydrolyze MBP in bipolar disorder may become an additional link between the processes of myelin damage and inflammation.

## 1. Introduction

The prevalence of bipolar affective disorder (BD) is about 1% in the general population [1] and has remained consistently high over the past 30 years [2]. Bipolar affective disorder is a chronic disorder of mood that is characterized by a combination of manic, hypomanic and depressive episodes, which are expressed as recurrent cases of changes in energy levels and behavior [3]. It can be subdivided into bipolar disorder I (BD I), defined by the occurrence of at least one lifetime manic or mixed affective episode along with and recurrent depressive episodes and bipolar disorder II (BD II), defined by the occurrence of at least one hypomanic episode and one major depressive episode [4]. This disorder is associated with high rates of premature mortality from medical comorbidities and suicide [5]. However, the etiology and mechanisms underlying bipolar disorder are not well understood. Numerous diffusion tensor imaging studies [6], genome-wide association studies [7,8], and postmortem studies [9,10] have demonstrated a reduction in myelin content in the CNS, aberrant expression of myelin-related genes and damage of oligodendrocytes, along with decreased myelin staining in BD patients. A recent meta-analysis involving 32 studies confirms a reduction in fractional anisotropy and an increase in radial diffusivity in individuals with BD compared to healthy subjects, supporting the hypothesis of alterations in myelination as a possible mechanism of BD [6]. Myelination in BD might change after affective episodes [11], accompanied by altered activity levels or cognitive biases associated with depression and mania [12]. Additionally, a global age-related deficit in intracortical myelin maturation throughout the cortex has been identified in bipolar disorder, which the authors of the study attribute to potential cognitive and behavioral impairments [13].

One of the main protein components of myelin in the human CNS is myelin basic protein (MBP), which may enter the cerebrospinal fluid or bloodstream and have highly immunogenic properties [14]. MBP is responsible for the adhesion of myelin sheaths of oligodendrocytes, may participate in signaling in the nucleus, in interactions with the cytoskeleton, and a regulatory function in the expression of other myelin proteins [15]. Antibodies to MBP appear in violation of the integrity of myelin during stroke [16], brain injury [17], multiple sclerosis [18], systemic lupus erythematosus [19], autism [20], and schizophrenia [21].

It is suggested that immune system dysfunction and inflammatory processes may influence myelination abnormalities in various psychiatric disorders, including BD [22,23,24]. The integrity and permeability of the blood–brain barrier change during pro-inflammatory conditions during bipolar depression [25]. Recent studies have shown that bipolar disorder involves microglial activation in the hippocampus and alterations in peripheral cytokines, suggesting a potential link between neuroinflammation and peripheral toxicity [24,26]. There is evidence of a reduced number of circulating T-regulatory lymphocytes, which suppress hyperactive immune responses and the development of autoimmune diseases [24,26], and their correlations with brain white matter integrity in patients with BD [22,23]. Furthermore, recent works have thoroughly supported the association between BD and a proinflammatory state, involving both the innate and the adaptive immune system. In particular, they revealed an increase in the production of IL-4, IL-5, and IL-10 suggests Th2 cells’ activation and enhanced antibody-mediated reactions [13]. A wide range of autoantibodies were identified in BD, including a group of antinuclear and antiphospholipid autoantibodies, antibeta2-glycoprotein, anti-tissue transglutaminase IgA [27], and anti-thyroid peroxidase antibodies [28]. In addition, autoantibodies to neuro-specific antigens, such as IgM to NMDA receptors, have been found in cases of bipolar affective disorder and psychosis [29,30], including the early stages of the disease [30]. It is reasonable to assume that among such a wide range of antibodies in BD, antibodies with catalytic activity may also appear.

Over recent decades, the catalytic properties of antibodies have been demonstrated, which allow them not only to bind but also to hydrolyze antigens [31,32,33]. Natural and artificial antibodies with catalytic functions are also called abzymes. Antibodies hydrolyzing myelin basic protein have been found and described in detail in multiple sclerosis (MS) [34], systemic lupus erythematosus (SLE) [35], autism [36], and schizophrenia [37]. We also showed earlier pronounced differences in the level of MBP-hydrolyzing activity of serum antibodies depending on the clinical features of schizophrenia, such as the type of course of the disease, the duration of the disease, and the presence of remission [38]. In addition, the cross-reactivity of antibodies with histone-hydrolyzing and MBP-hydrolyzing activity, as shown in a series of studies [39,40,41,42], indicates a connection between inflammation and damage of the myelin sheath.

The first work on the study of catalytic antibodies in bipolar disorder appeared in 2021, indicating the presence of DNA-hydrolyzing activity of serum IgG [43]. However, antibodies with proteolytic activity against neurogenic proteins, such as myelin basic protein (MBP), have not been previously studied in patients with BD. Taken together, data on abnormalities of the white matter of the brain in BD associated with immunological disorders and a decrease in the content of MBP form the basis for the study of the MBP-hydrolyzing activity of antibodies in BD.

This paper presents the first evidence of the presence of MBP-hydrolyzing properties in serum IgG from patients with bipolar disorder, and also provides a detailed characterization of their activity, including kinetic parameters, pH dependence, and inhibitory analysis.

## 2. Results

### 2.1. Characterization of Patients and Healthy Donors

In this work, we recruited patients with bipolar disorder as the main study group and healthy donors as the control group. In previously published studies, we have already presented evidence of the presence of MBP-hydrolyzing antibodies in patients with schizophrenia and their heterogeneity [37,38]. In this work, patients with schizophrenia were presented as the comparison group. The patients with bipolar disorder and healthy individuals were comparable in age (*p* = 0.54; *t*-test), but there was a slight difference in the sex composition. Patients with bipolar disorder and schizophrenia were comparable in age (*p* = 0.54; *t*-test) and disease duration (*p* = 0.45; *t*-test). Control subjects were mentally and somatically healthy individuals. Since the exclusion criteria for all groups included the presence of acute and chronic infectious, inflammatory, autoimmune, or neurological diseases, and organic brain disorders, all these factors could not affect the results of the study. The influence of drug therapy was excluded by taking blood from patients during hospitalization before starting medication. More detailed characteristics of the patients and healthy donors are presented in Section 4.2 and Table 1.

### 2.2. Antibody Purification and Application of Strict Criteria for IgGs with Proteolytic Activity

**Figure 1:**
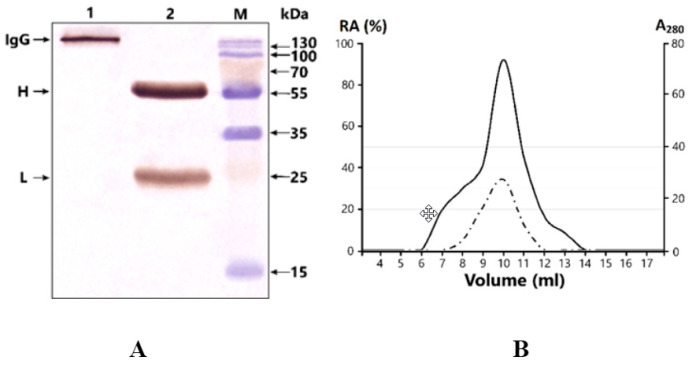
(**A**) SDS-PAGE analysis of homogeneity IgG in 12.5% gel followed by silver staining. Lane 1—intact IgG; lane 2—IgG incubated with 40 mM DTT at 100 °C (conditions for the complete reduction of disulfide bonds); lane M—protein molecular weight markers. (**B**) FPLC gel filtration on Superdex200 column of IgGmix in an acidic buffer (100 mM Gly-HCl, pH 2.6) after IgG preincubation in the same buffer. Absorbance at 280 nm (A280); the relative activity (%) of the obtained fractions in the hydrolysis MBP.

IgG was isolated by affinity chromatography from the serum of patients with bipolar disorder and healthy individuals on Protein-G Sepharose under conditions that remove non-specifically bound proteins. This method has been used many times to purify electrophoretically homogeneous IgG preparations [34,37,39,42]. Subsequent homogeneity checks in 4–18% SDS-PAGE and silver staining after transfer to the PVDF membrane showed only a single band, corresponding to 150 kDa, under nonreducing conditions, and two bands of the H and L chains after reduction (Figure 1A). No additional bands were found to reflect the presence of other proteins.

The fast protein liquid chromatography (FPLC) gel filtration of IgGmix under conditions of “acidic shock” (pH 2.6) resulted in the elution of 17 fractions, with a single pronounced peak of optical density corresponding exactly to 150-kDa IgGs. The subsequent evaluation of the activity in the isolated fractions demonstrated a complete correspondence between the profile of the optical density A280 and the profile of the MBP-hydrolyzing activity of IgGmix (Figure 1B). This indicates that homogeneous IgGs exhibit MBP hydrolyzing activity and did not contain any impurities of other proteases.

**Figure 2:**
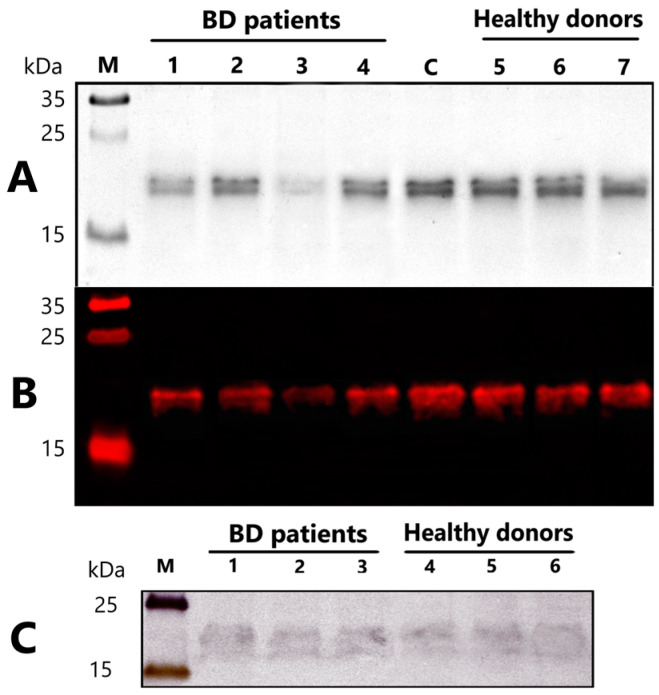
(**A**). SDS-PAGE analysis of the proteolytic activity of IgG in the hydrolysis myelin basic protein (MBP). (**B**). Immunostaining of reaction mixture after SDS-PAGE with primary anti-myelin basic protein antibody. The reaction mixtures containing 1 mg/mL MBP and 20 mM Tris-HCl (pH 7.5) were incubated for 20 h at 37 °C in the presence of 0.2 mg/mL IgG. Lanes 1–4—MBP after incubation with individual IgGs of patients with bipolar disorder (BD); lanes 5–7—MBP after incubation with individual IgGs of healthy donors; lane C—control reaction mixture without IgG; lane M—protein molecular weight markers. (**C**). Western blotting of MBP-binding IgG with primary anti-human IgG antibody. Lines 1, 2, 3–PVDF membrane with MBP after incubation in an equimolar mixture of three IgG from bipolar patients; lines 4, 5, 6—PVDF membrane with MBP after incubation in an equimolar mixture of three IgG from healthy donors; M—protein molecular weight markers.

The analysis of MBP-hydrolyzing activity was investigated by the separation of products in 12.5% SDS-PAGE. Analysis of the profile of MBP-hydrolysis was performed with individual antibodies of patients with BD and healthy donors. In Figure 2, the SDS-PAGE analysis (A) followed by immunostaining using anti-myelin basic protein antibody (B) is shown. The analysis demonstrates that the incubation of an MBP with IgG of BD patients led to a decrease in the intensities of stained initial MBP bands. The incubation of the substrate and IgG of healthy individuals did not lead to a decrease in the intensity of the initial MBP band. At the same time, purified IgG of BD patients, as well as of healthy donors, contains fraction of MBP-binding antibodies, as shown with Western blotting (Figure 2C). The band with MBP after transfer to the PVDF membrane is located between 15 kDa and 25 kDa. After staining with anti-human IgG antibody, the stained purified polyclonal IgG are located in the same molecular weight region. There was no pronounced difference in the intensity of MBP-binding IgG’s bands in patients and healthy individuals.

### 2.3. The Catalytic Properties of MBP-hydrolyzing IgGs in Bipolar Disorder

**Figure 3:**
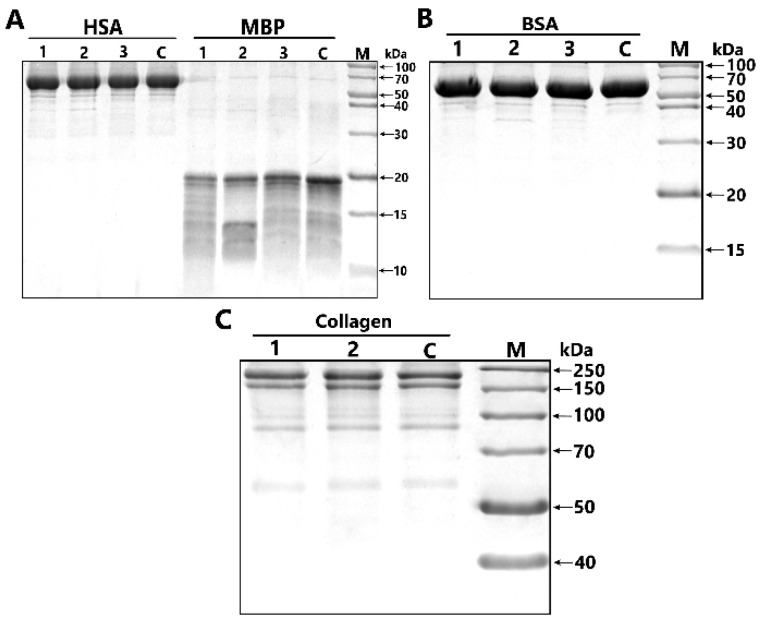
SDS-PAGE analysis of substrate specificity of three different IgG-1, IgG-2 and IgGmix (lanes 1, 2 and 3, respectively) of patients with bipolar disorder in the hydrolysis of the following various proteins: (**A**) human serum albumin (HSA), myelin basic protein (MBP), (**B**) bovine serum albumin (BSA), (**C**) collagen. In all cases, lane C corresponds to various proteins incubated in the absence of IgG, and lane M corresponds to protein molecular weight markers (**A**–**C**).

The analysis of substrate specificity demonstrated that the IgGs of two different BD patients and IgGmix (an equimolar mixture of five IgG from five different patients) effectively hydrolyzed MBP, but did not hydrolyze the other tested proteins (human and bovine serum albumin and rat collagen) under the same reaction conditions (Figure 3). Thus, the substrate specificity of MBP-hydrolyzing IgGs of BD patients was shown; in contrast, canonical proteases efficiently hydrolyze a variety of proteins.

**Figure 4:**
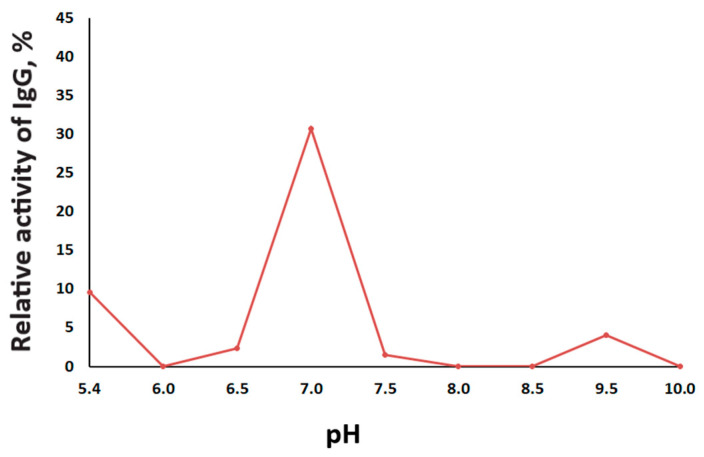
The pH dependence of the relative proteolytic activity of IgGmix in the hydrolysis of MBP. The relative proteolytic activity corresponding to complete hydrolysis of MBP after 20 h of incubation with 0.2 mg/mL IgGmix was taken as 100%.

Analysis of the pH-dependence of the MBP-hydrolyzing activity was performed for the total IgGmix of five patients with high and medium activity (Figure 4). In our study, analysis of MBP-hydrolyzing activity of IgGs of BD patients was carried out within a wide range of pH values 5.4–10.0, considering MBP stability in the pH range of 4–10.5 [44]. Catalytic IgGs of BD patients are able to efficiently hydrolyze MBP within a wide range of pH values 5.4–9.5, with a pronounced optimum of MBP hydrolysis at pH 7.0. Additionally, less pronounced peaks of proteolytic activity were registered at pH 5.5 and pH 9.5.

**Figure 5:**
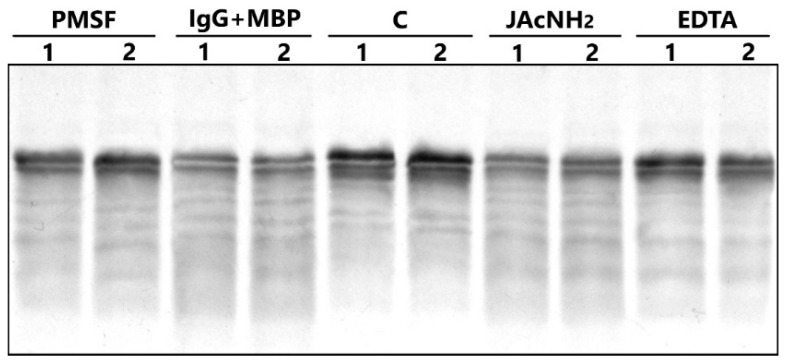
SDS-PAGE analysis of the hydrolysis of MBP by IgG in absence and presence of different inhibitors. Lanes 1 and 2 correspond to two repeats. The mixtures containing 1 mg/mL MBP and 0.2 mg/mL IgG were incubated for 24 h. Lanes C—control reaction mixture containing MBP and 20 mM Tris-HCl (pH 7.5) without IgG; lanes IgG + MBP—mixture of IgG and MBP without inhibitors; lanes PMSF, JAcNH2, EDTA—mixture of IgG and MBP in the presence of relevant inhibitors.

An analysis of the type of proteolytic activity was performed with IgGmix of BD patients in the presence and the absence of specific inhibitors. The IgG-dependent hydrolysis of MBP after 24 h of incubation led to the cleavage of 43% of the substrate compared with the control (lane C without IgG) (Figure 5). Preincubation of IgGmix with iodoacetamide (the specific inhibitor of thiol proteases) very poorly protects MBP from hydrolysis by IgGmix (13%). At the same time, PMSF (inhibitor of serine proteases) and EDTA (inhibitor of metalloproteases) significantly suppressed the proteolytic activity of IgGs of patients with BD at 43% and 68.2%, respectively.

**Figure 6:**
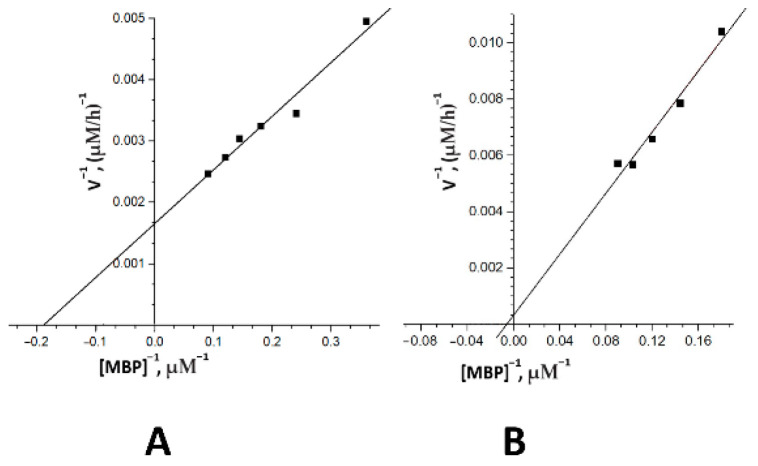
Determination of the kinetics parameters of MBP hydrolysis using the Lineweaver–Burk plot in the case of two individual IgG-1 (**A**) and IgG-2 (**B**) preparations of two patients with bipolar disorder.

We have estimated the Km and kcat values for the hydrolysis of MBP for two individual IgGs of BD patients using the Michaelis–Menten approach and Lineweaver–Burk coordinates (Figure 6). The following two IgG preparations with pronounced activity were selected for the study: IgG-1 (male, 1.5 years of illness, BD I type (F31.6), with 4 episodes) and IgG-2 (female, 16 years of illness, BD II type (F31.31), with 12 episodes). The dependence of the MBP hydrolysis rate on the substrate concentration for two IgG preparations confirmed the Michaelis–Menten kinetics (Figure 6). The initial rate data obtained at increasing MBP concentrations were consistent with the Michaelis–Menten kinetics (Figure 3). The affinity of MBP (in terms of Km values) for two different IgGs varied from 5.2 to 150 mkM, while the apparent kcat ranged from 3.85 × 10^−6^ to 7.7 min^−1^. The results are summarized in Table 2.

### 2.4. Comparison of IgGs MBP-Hydrolyzing Activity of Healthy Individuals and Patients

The relative activity of IgGs in the cleavage of MBP was estimated from the decrease in the intensity of the Coomassie-stained MBP band after electrophoresis, according to the literature [34,35]; the difference in the intensities of the MBP incubated in the absence and presence of IgGs was used for the correction of the values.

**Figure 7:**
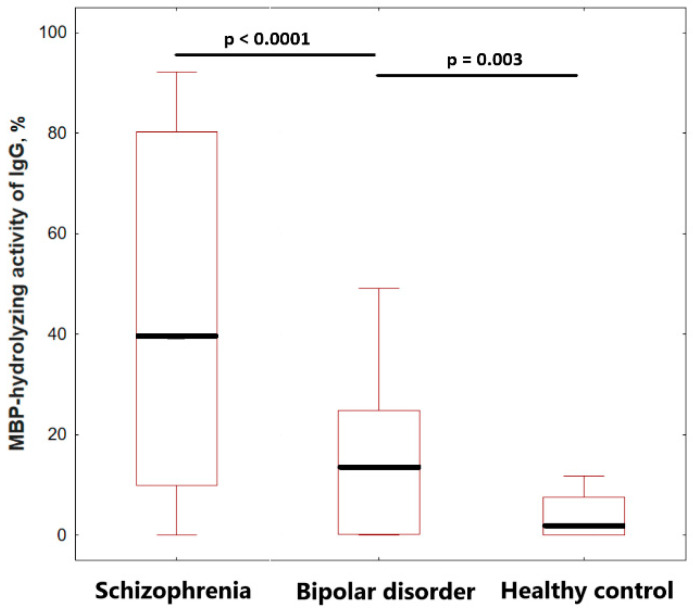
The MBP-hydrolyzing activity of IgGs from the sera of patients with bipolar disorder, schizophrenia and healthy peoples. The level of MBP-hydrolyzing activity of IgGs was normalized to standard conditions (1 mg/mL MBP proteins; 0.2 mg/mL IgGs; 20 h of incubation at 37 °C). The complete hydrolysis of the MBP as substrate is taken as 100%. The significance of the differences (*p*) is calculated by the Mann–Whitney U test.

The screening results showed that the relative activity level of MBP-hydrolyzing antibodies varied significantly from patient to patient in a range from 0 to 49.1%. Analysis of the level of MBP-hydrolyzing activity of IgG in patients with bipolar disorder revealed a statistically significant (*p* = 0.003) increase in activity by 1.6 times, compared with healthy individuals (Figure 6). The median values of the MBP-hydrolyzing activity level were 9.33 (0.23; 25.46) in bipolar disorder and 5.71 (0.00; 9.88) in the healthy control group. A comparative analysis revealed a significant (*p* < 0.0001) 3.5-fold decrease in the specific activity of IgG in patients with bipolar disorder, compared to in patients with schizophrenia, with 37.40 (9.00; 87.00%) (Figure 7).

Patients with bipolar disorder showed different proportions of antibodies with low (up to 10%), medium (from 10% to 40%), and high activity (more than 40%). Among the 25 IgG preparations of patients with bipolar disorder, 48% (12 people) were low-active antibodies, 44% (11 people) were antibodies with medium activity and only 8% (2 people) had high activity. In schizophrenia, the proportion of IgG with medium activity was 23.1% (12 people), and highly active IgG was 46.2% (18 people) among 30 patients. Thus, there is significant heterogeneity in both the individual level of activity of MBP-hydrolyzing antibodies and the spectrum of activity in bipolar disorder and schizophrenia.

We did not find significant differences in the level of MBP-hydrolyzing activity in groups with moderate or severe depressive episodes. The statistical analysis did not reveal significant differences (*p* < 0.053); however, the 5-fold difference in the proteolytic activity level of antibodies in patients with a disease duration less than 5 years (24.07%) and more than 5 years (4.98%) may indicate a tendency to reduce catalytic activity. We did not find any statistically significant correlation with the duration of the disease, the age of manifestation, or with the age of the patients. The statistical analysis did not show a significant correlation of MBP-hydrolyzing activity levels with scores according to the SIGH-SAD scale, CGI-10, HCL-32 or number of episodes. The results of the correlation analysis are presented in detail in Appendix A (Table A1).

## 3. Discussion

In this work, we discovered antibodies hydrolyzing myelin basic protein from the serum of patients with bipolar disorder and described their biochemical properties. The combination of strict criteria applied in this study allows us to prove that the catalytic properties belong to purified antibodies. In our work, the following were used: isolation on an affinity sorbent, assessment of the homogeneity of IgG preparations, gel filtration under acid shock conditions to separate non-covalent complexes, and substrate specificity. Therefore, the catalytic activity detected in the further stages of the study can be attributed directly to isolated IgG and not to potentially co-obtained proteases.

Studies of antigen affinity of MBP-hydrolyzing antibodies that were performed with chromatography on MBP-Sepharose show that the MBP-binding fractions of polyclonal IgGs are able to hydrolyze it specifically [35,45,46]. It was shown that intact MBP interacts with variable parts of heavy and light chains of immunoglobulins, but its catalytic centers are located on the light chain, while the heavy chain is mainly responsible for specific antigen recognition and increased antigen affinity for antibodies [47]. Since in our work we used polyclonal IgGs, we suggest that MBP-hydrolyzing properties correspond to the anti-MBP antibodies. The results of the substrate specificity analysis and Western blotting of MBP-binding IgG indirectly confirmed that suggestion.

The characterization of the catalytic properties of abzymes with MBP-hydrolyzing activity in BD was presented for the first time, revealing their high heterogeneity and significant differences from canonical proteases.

It has been previously shown that MBP-hydrolyzing abzymes can be a mixture of IgG proteases with different types of proteolytic activity, including serine, acid, thiol, or metal-dependent proteases, the proportion of which can vary in different diseases. For example, the proteolytic activity profile of IgG in patients with schizophrenia includes hydrolysis mechanisms similar to thiol proteases (suppression of the initial level of activity by 22–78.6% with iodoacetamide), serine proteases (suppression by 73–100% with PMSF), and metal-dependent proteases (suppression by 24–98% with EDTA) [37]. At the same time, the proteolytic activity of IgGs from MS and SLE patients is not very sensitive to iodoacetamide (the range of inhibition is only 5–15%), but they have a large proportion of serine proteases (30–70% of inhibition in patients with MS and 39–42% in SLE patients) [34]. EDTAs decrease the MBP-hydrolyzing activity of IgGs to a greater extent in SLE (50–98%) than in MS (10–80%), which indicates the proportion of metalloproteinases [35]. Thus, the activity spectrum of MBP-hydrolyzing IgGs in BD is significantly different from the previously described abzymes.

Analysis of the pH-dependence of the MBP-hydrolyzing activity of abzymes in SLE, multiple sclerosis, and schizophrenia showed high heterogeneity of the pH optimum and individual variability for each patient. This is in contrast to most classical proteases with a pronounced pH optimum [48]. According to our previous data, the MBP-hydrolyzing activity of IgG in schizophrenia is increased in a pH range between 6 and 8.5, with an optimum at pH 7.5, and at acidic pH values, the activity is reduced. For patients with SLE, maximum IgG activity was observed in the pH range of 7.5–8.5 [35]. At the same time, two maximums of proteolytic activity at pH 6.0 and 9.5 were observed in MS, while pronounced hydrolysis of MBP was observed under acidic conditions, including individual peaks at pH 2.6, 4.2, and 5.4 [49]. Thus, some proteolytic abzymes exhibit properties of acid proteases, such as MBP-hydrolyzing IgG in MS or histone-hydrolyzing IgG in schizophrenia [39]. This is comparable to lysosomal enzymes having activity in the foci of inflammation [49,50,51]. The hydrolysis of MBP by IgGs of BD patients proceeded at very different pH values from 5.4 to 9.5, including a pH optimum at 7.0 and additional areas of pronounced activity at pH 5.4 and 9.5. Since we used polyclonal serum IgGs in this work, it is possible to assume that MBP-hydrolyzing abzymes in BD are heterogeneous and could consist of different sets of catalytic IgG subfractions with different pH dependencies. It is likely that this heterogeneity allows abzymes to perform efficient catalysis both under peripheral blood pH conditions and under the acidic pH of the inflammation focus [52,53].

Analysis of the kinetic parameters of the antibody-mediated MBP-hydrolysis showed that the Km values (5.2–150 mkM) of IgG-dependent proteolytic activity are relatively higher in BD patients in comparison with MS patients (0.9–5.0 mkM) [49], SLE patients (0.6–2.7 mkM) [35], schizophrenia patients (4.3–12.4 mkM) [37] and canonical proteases. The Km values describe the affinity of the enzyme to the substrate. Thus, the affinity of IgGs in BD is lower compared to previously identified MBP-hydrolyzing IgGs of patients with MS and SLE. A higher than 30-fold decrease in IgG affinity in patients with BD following increased disease duration was observed in patients with 1.5 years and 16 years of disease duration. Antibodies against protein antigens may undergo affinity maturation over months or years upon repeated antigen presentation [54,55], which may occur in the inflammation response during recurrent episodes of BD. Interestingly in our case, catalytic antibodies showed a reverse change in affinity. It is known that the rate of catalysis (k_cat_) of abzymes varies in the range of 10^−6^ to 40 min^−1^ in autoimmune disorders [34,35,49,56]. We also found a wide variation in k_cat_ (3.85 × 10^−6^–7.7 min^−1^) in the case of IgG patients with bipolar disorder. The highest k_cat_ values were found for the IgG-1 antibody with a lower affinity for the substrate.

A pro-inflammatory status, neuroinflammation, and high comorbidity with autoimmune disorders can become a biological background for the generation of catalytic antibodies in bipolar disorder. Thus, a meta-analysis of 34,633 patients in 2021 showed high comorbidity of bipolar and autoimmune diseases (95% CI, 1.62–3.07), as well as a significantly higher incidence of bipolar disorder among patients with AD, compared with patients without them (95% CI, 1.28–1.86) [57]. A reduced number of circulating regulatory T lymphocytes, which suppress erroneous immune responses, indicates immune/inflammatory imbalance in patients with BD [54,58]. The activated inflammatory response system, including inflammation-related serum cytokines (TNF-α, IL-8, IFN-γ, and IL-10), growth factors (IGFBP2, PDGF-BB), and reduced circulating Treg lymphocytes, has significant associations with white matter integrity [23,26] and gray matter volume [59]. However, the mechanisms of damage to the myelin sheath in BD are still the subject of research. The ability of serum IgG to hydrolyze MBP in bipolar disorder, which we have revealed, may become an additional link between myelin damage and inflammation.

Our study of the proteolytic activity of antibodies revealed a significant (*p* < 0.0001) decrease in the MBP-hydrolyzing activity of IgG in bipolar affective disorder, compared to in schizophrenia. The low substrate affinity and levels of proteolytic activity indicate that IgG-dependent hydrolysis of MBP is less effective in bipolar disorder compared to in schizophrenia. A comparison of the level of MBP-hydrolyzing activity in autoimmune and mental disorders showed that the percentage of antibodies with pronounced activity decreases in the following series: MS (84%) < schizophrenia (69%) < BAR (52%) [34,37]. Our results are consistent with the first study of DNA-hydrolyzing IgG in bipolar disorder, which showed a decrease in activity levels compared with patients with schizophrenia and SLE [43]. The differences in the level of proteolytic activity of serum antibodies revealed in this article indirectly indicate differences in the pathogenesis of affective and psychotic disorders, when autoimmunity can be much less pronounced in bipolar disorder than in schizophrenia.

In BD patients, we observed a tendency towards a decrease in the level of proteolytic activity with disease duration over 5 years (*p* < 0.053), along with a decrease in the affinity of MBP-hydrolyzing antibodies, according to the analysis of the kinetic parameters. This is a notable difference in MBP-hydrolyzing antibodies in BD from schizophrenia, in which both proteolytic activity [37] and hypomyelination of the cortical and white matter of the brain increase with the duration of the disease [60].

Notably, a study on the levels of around 17 different serum antinuclear and antiphospholipid autoantibodies via ELISA among 585 patients with psychotic and affective disorders found no specific differences [27]. Additionally, no difference was found between patients with mood disorders and psychotic disorders in the study of the level of cytokines in the CSF [61], myelin basic protein in the CSF [58], as well as serum inflammatory markers [62]. In this context, it can be assumed that in some cases, the catalytic activity of antibodies may be a more informative indicator for identifying differences between affective and psychotic disorders than the concentration of serum analytes. In our study, the results of Western blotting analysis also did not reveal a pronounced difference in the content of MBP-binding IgG between patients with bipolar disorder and healthy individuals. However, these results are consistent with data on DNA-binding and DNA-hydrolyzing IgGs in BD. Ramesh R. et al. showed that the DNA-hydrolyzing activity of antibodies is increased in patients with bipolar disorder compared to healthy individuals, but the titers of antibodies to DNA are even lower than in healthy subjects [43].

There are two main views on the pathophysiological role of catalytic antibodies in various diseases [32]. One line of evidence suggests that proteolytic abzymes may have a protective function, promoting the breakdown of pathogenic proteins and elimination of antigens from the peripheral circulation, as suggested for amyloid-hydrolyzing abzymes in Alzheimer’s disease [63]. MBP fragments can enter through the damaged blood–brain barrier [25] into the peripheral circulation and stimulate the formation of autoantibodies, including antibodies with proteolytic activity.

Another line of evidence suggests a negative role for abzymes associated with possible cross-reactivity between histone-hydrolyzing antibodies and MBP-hydrolyzing antibodies, as has been shown in SLE [41], and HIV-infected [64] patients. It is suggested that due to low-level inflammation and tissue damage, histone-hydrolyzing antibodies can be formed, which can also hydrolyze the myelin basic protein. In BD, transient or persistent loss of BBB integrity is associated with decreased CNS protection and increased permeability of proinflammatory (e.g., cytokines, reactive oxygen species) substances from the peripheral blood into the brain [25]. Furthermore, inflammatory cytokines, which are present in the CNS or blood during neuroinflammation, affect the stability of the BBB by degrading the tight junctions’ proteins between endothelial cells, thus allowing IgG to enter the CNS from the bloodstream or cerebrospinal fluid [65]. It is likely that in this case, MBP-hydrolyzing IgG can damage the myelin sheath of axons, similarly to in multiple sclerosis, thus violating the integrity of neuronal circuits.

There are some limitations of our study. The small sample size in this study did not allow for a detailed assessment of clinical subgroups, including the type of bipolar disorder; this likely affected the results of the correlation analysis of proteolytic activity with clinical data. Since the majority of patients had a long period of illness, we cannot completely exclude the influence of drug therapy on the obtained results. However, to minimize their impact, the design of the study involved the sampling of biomaterial before starting medications. Hypertension was not included in the exclusion criteria list, which possibly could affect our results for some participants because of the association between hypertension and inflammation. Individuals from the control group and patients’ group did not undergo a brain MRI study, which limits the evaluation of the pathophysiological effect of MBP-hydrolyzing antibodies on myelin.

Bipolar disorder is known to be strongly associated with inflammation [66,67,68,69]; this is indicated by peripheral markers, ref. [70,71], the neuroinflammatory status of brain tissue [72,73] and genetic studies [74]. There is also an association between the clinical features of the disease and indicators of inflammation [75,76]. The ability of serum IgG to hydrolyze MBP in bipolar disorder, which we have revealed, may become an additional link between the processes of myelin damage and inflammation in bipolar disorder. However, further investigations of immunological disturbances disorders and their influence on catalytic antibody production are needed to explain these mechanisms.

## 4. Materials and Methods

### 4.1. Patients, Healthy Donors, and Biological Material

In this work, 25 patients (29% males/71% females, mean age = 37.1 ± 14.6 years) with bipolar disorder and 20 healthy donors (42% males/58% females, mean age = 34.96 ± 10.13 years) were recruited to study the proteolytic activity of their antibodies. Among the 25 patients, 20 individuals had BD I type and 5 individuals had BD II type. As a comparison group, we presented data on 30 patients with paranoid schizophrenia (31% males/69% females, mean age = 39.42 ± 11.5 years). The schizophrenia diagnosis was confirmed and verified in accordance with the international standard criterion, the psychometric PANSS scale. Patients were recruited using the continuous method at the Mental Health Research Institute (MHRI) of the Tomsk National Research Medical Center (TNRMC; Tomsk, Russia).

Clinically, the verification of diagnoses was provided according to the International Classification of Diseases, 10th revision (ICD-10); the overall clinical assessment of the severity of bipolar disorder was assessed using the CGI scale (Clinical Global Impression) by psychiatrists from the Department of Affective States in the MHRI. The severity of current depression was assessed using the Hamilton Depression Scale, Seasonal Affective Disorder Version (SIGH-SAD). The Hypomania Check List (HCL-32) were used to identify lifetime history of hypomanic symptoms. Data on the participants are presented in Table 1. Inclusion criteria for the study were the following: bipolar disorder according to the International Statistical Classification of Diseases and Related Health Problems, 10th Revision (ICD-10: F31.3, F31.4, F31.6, F31.8), and the Structured Clinical Interview for DSM-IV Axis I Disorders (SCID). Exclusion criteria were the following: the presence of acute or chronic infectious, inflammatory, autoimmune or neurological diseases, type 2 diabetes, obesity, other biological mental disorders, and intellectual disability. Detailed characteristics of patients and healthy donors are presented Table 1.

Individuals included in the study gave written informed consent. Ethical approval was granted (protocol number 147/4.2021) by the Local Bioethics Committee of the Mental Health Research Institute of the Tomsk National Research Medical Center (Tomsk, Russia), in accordance with Helsinki ethics committee guidelines. None of the participants were compromised in their capacity/ability to consent; thus, consent from the next-of-kin was not necessary, and was not recommended by the local ethics committee.

Blood sampling was carried out before prescribing any treatment. Vacuette tubes with coagulation activators were used for fasting venous blood sampling after an overnight fast. To isolate the serum, the blood samples were centrifuged for 30 min at 2000× *g* at 4 °C. The sera were stored at −80 °C until analysis.

### 4.2. Purification of Serum IgGs

Immunoglobulins from the blood sera of patients and healthy controls were individually purified by earlier developed procedures for the purification of electrophoretically and immunologically homogenous IgG preparations from human blood serum [34,35,37,77]. Purification of IgGs from blood serum was carried out by affinity chromatography on Protein G-Sepharose using an ÄKTA pure chromatography system (GE Healthcare Bio-Sciences, Sweden), followed by high-performance gel filtration on a Superdex-200 HR 10/30 column. This method allows for selective elution of components of the immune complexes under conditions with increased ionic strength, without destroying the Ig complexes with Protein G [78]. Elution of IgG was carried out using 100 mM Gly-HCl buffer, pH 2.6, after which the resulting sample was immediately neutralized with 1 M Tris-HCl buffer, pH 8.8. Subsequent homogeneity analysis was performed under 4–18% SDS-PAGE for both native IgGs and separated heavy and light chains after incubation with DTT for the reduction of disulfide bonds.

### 4.3. High-Performance Gel Filtration of Antibodies under “Acid Shock” Condition

A mixture of five electrophoretically homogeneous preparations of IgG was preincubated in glycine buffer (pH 2.6), followed by gel filtration in the same buffer (acid shock) to separate the non-covalent complexes. The IgG was separated by high-performance gel filtration on a Superdex 200 HR column equilibrated with 50 mM glycine-HCl (pH 2.6) containing 0.1 M NaCl (buffer B), using Akta pure chromatography (GE). The eluate was sequentially collected in individual tubes as a separated fraction of the IgG preparation. After one week of storage at 4 °C, for refolding after the acid shock, the IgG was used in the activity assays as described below.

### 4.4. Proteolytic Activity Assay of IgGs

Evaluation of the proteolytic activity of serum IgG was performed using human brain MBP provided by the Department of Biotechnology, Research Center of Molecular Diagnostics and Therapy (Moscow). The reaction mixture (10 μL) for analysis of MBP-hydrolyzing activity of IgG contained 1 mg/mL MBP, 20 mM Tris-HCl (pH 7.5), and IgG in concentration 0.2 mg/mL, and was incubated at 37 °C for 5–24 h (standard reaction time was 20 h). The products of protein cleavage were analyzed in 4–15% or 12% SDS-PAGE according to the Laemmli method [39]. The polypeptides were visualized by silver or Coomassie R250 staining [79]. The catalytic activity of the IgG in the cleavage of MBP was estimated from the decrease in the intensity of the Coomassie-stained MBP band after electrophoresis. Differences in the hydrolysis levels of MBP incubated in the absence and in the presence of IgG were used to correct the values. Gels were visualized using the iBright Imaging Systems FL1500 gel documentation system (Thermo Scientific, Waltham, MA, USA) on base of the Core Facility “Medical Genomics” Tomsk NMRC. Quantitative evaluation of proteins was estimated using the iBright Analysis Software.

### 4.5. Analysis of Substrate Specificity of Antibodies

To study substrate specificity of IgG preparations of two different patients with BD and an equimolar mixture of five IgG preparations (IgGmix), they were incubated for 24 h at 37 °C under the same standard conditions (20 mM Tris-HCl pH 7.5, 1 mg/mL of protein substrates, 0.2 mg/mL IgGs) in the presence myelin basic protein, human and bovine serum albumin, and rat collagen. The analysis of hydrolysis products was carried out in 12% and 4–18% SDS-PAGE. The hydrolysis products after staining with Coomassie blue R-250 were registered as described above.

### 4.6. The Effect of pH on the Proteolytic Activity of IgG

The effect of pH on the activity of IgGs was evaluated using different 50 mM buffers, which are as follows: Gly-HCl buffer (pH 5.4–6.0), MOPS (pH 6.5–7.0), Tris-HCl (pH 7.5–9.0), and Gly-NaOH (pH 9.5–10.0). An equimolar mixture of five IgG preparations (IgGmix) was used for the analysis. The IgGs relative proteolytic activity (%), depending on the reaction mixture’s pH, was determined as described above. The time of incubation was 20 h at 37 °C.

### 4.7. Inhibitory Assay of MBP-hydrolyzing Activity

For analysis of a possible type of proteolytic activity of IgGs, an equimolar mixture of five IgG preparations (IgGmix) was pre-incubated for 30 min at 37 °C with one of the specific inhibitors of different proteases, including iodoacetamide (4.0 mM), PMSF (1.5 mM), or EDTA (50 mM), and aliquots of these mixtures were then added to the standard reaction mixtures, which were incubated for 24 h at 37 °C. The final concentration of IgGmix in the reaction mixture was 0.2 mg/mL. The initial level of proteolytic activity of IgGmix in the cleavage of MBP was estimated from the decrease in the intensity of the Coomassie-stained MBP band in the presence of IgGmix without inhibitors, as described above in 4.4. Then, the level of proteolytic activity of IgGmix in the presence of inhibitors was estimated and the percentage of inhibition relative to the initial level of activity was calculated.

### 4.8. Kinetic Parameter Analysis

The study of the kinetic parameters was carried out in 20 mM Tris-HCl (pH 7.5) at a fixed concentration of IgG 0.2 mg/mL (1.3 mm), and the following concentrations of MBP: 0.05, 0.075, 0.1, 0.125, 0.15, 0.175, and 0.2 mg/mL. The products of the MBP hydrolysis were analyzed using SDS-PAGE, as described above. The specific activity was calculated via the iBright Analysis Software using the substrate loss due to hydrolysis. The kinetic constants were then determined using non-linear approximation by the least-square method in the Origin 9.0 program (OriginLab Corporation, Northampton, MA, USA); the kinetic data were presented as Lineweaver–Burk coordinates.

### 4.9. Immunoblotting of Myelin Basic Protein

The standard reaction mixture after incubation with IgG of BD patients or healthy individuals was separated on SDS-PAGE, as described above in Section 4.4 and transferred to polyvinylidene difluoride (PVDF) membranes using electroblotting in tris glycine transfer buffer. The blot was pre-incubated for 1 h in a blocking buffer (5% BSA in phosphate-buffered saline (PBS)), then in primary anti-myelin basic protein (MBP) antibodies overnight at 4 °C at concentrations of 1:2000 (Aves Labs, Davis, CA, USA). The blot was washed with PBS containing 0.05% Tween (Sigma Aldrich, Saint Louis, MO, USA) (3 × 10 min), and incubated for 1 h with the secondary antibody conjugated with Alexa Flour 594 (1:2000, Jackson ImmunoResearch, West Grove, PA, USA). The blot was washed again in PBS-Tween (3 × 10 min) and the bands were visualized using the iBright Imaging Systems FL1500 (Thermo Scientific, Waltham, MA, USA).

### 4.10. Western Blotting of MBP-Binding IgG

Human MBP (5 µg) in six lanes was separated on 12.5% SDS-PAGE and transferred to PVDF membranes using electroblotting in tris glycine transfer buffer. The membrane was split into two parts for separate incubation with antibodies from healthy donors and antibodies from patients with bipolar disorder. The blots were pre-incubated for 1 h in a blocking buffer (5% BSA in PBS). Then, the membranes were incubated in an equimolar mixture of three purified IgG (0.3 mg/mL in PBS) from sera of healthy or bipolar patients overnight at 4 °C. The blot was washed with PBS containing 0.05% Tween (3 × 10 min) and incubated in primary anti-human IgG (Fc specific) antibodies (Sigma Aldrich, USA) at 4 °C at concentrations of 1:2000 for 3 h. The blot was washed with PBS containing 0.05% Tween (3 × 10 min) and incubated for 1 h with the secondary antibodies conjugated with HRP (1:5000). The blot was washed again in PBS-Tween (3 × 10 min) and stained with the Opti-4CN Substrate Kit (Bio-Rad, Hercules, CA, USA). The bands were visualized using the iBright Imaging Systems FL1500.

### 4.11. Statistical Analysis

Statistical analyses were performed using the Statistica 12.0 software for Windows. The Shapiro–Wilk test, used to check the normality of the distribution of data, demonstrated that most of the data did not meet the normal Gaussian distribution. For this reason, the differences between IgG samples of different groups were estimated using the Mann–Whitney test and the Kruskal–Wallis Test. For the correlation analysis, the non-parametric Spearman rank method was applied. Differences between the studied groups were considered statistically significant if *p* < 0.05. Data are presented as mean and standard deviation, as well as median (M) and quartiles (Q1; Q3).

## Figures and Tables

**Table 1:** Demographic and clinical characteristics of patients and healthy control subjects.

Parameter	Healthy Controls	Bipolar Disorder	Schizophrenia
Number of participants, persons	20	25	30
Sex (male/female), %	42/58	29/71	31/69
Age, years	34.96 ± 10.13	37.1 ± 14.6	39.42 ± 11.5
BD I type/BD II type, persons	-	20/5	-
Duration of disorder, years	-	12.1 ± 9.0	13.6 ± 7.59
Number of episodes	-	6.7 ± 5.0	-
CGI	-	4.6 ± 0.9	4.8 ± 0.7
SIGH-SAD total	-	24.4 ± 7.8	-
HCL-32	-	17.7 ± 4.7	-
PANSS total score	-	-	89.00 (80.00; 105.00)

**Table 2:** The kinetic parameters characterizing efficiency of MBP hydrolysis by two individual IgG preparations of patients with bipolar disorder.

Kinetics Parameters	IgG-1 ^1^	IgG-2 ^1^
Km, mkM	5.2 ± 0.43	150 ± 10.6
kcat, min^−1^	7.7 ± 0.35	(3.85 ± 0.9) × 10^−6^

^1^ The average values are reported as mean ± SD; at least three measurements were taken for each value; the measurement inaccuracy of reported values did not exceed 10–25%.

## Data Availability

The data presented in this study are available on request from the corresponding author.

## Appendix A

**Table A1:** Correlation analysis of MBP-hydrolyzing activity of IgGs of bipolar disorder patients with clinical data.

Parameter	Spearman Correlation Coefficient (r) *
Age, years	0.045
Age of manifistation, years	−0.003
Duration of disorder	−0.193
Number of episodes	−0.031
CGI	0.278
SIGH-SAD total	−0.297
HCL-32	−0.035

* Spearman correlation coefficients (r) were considered significant at *p* < 0.05; no significant differences were found.
